# Supplementary material for: Metabolic Dysregulation Explains the Diverse Impacts of Obesity in Males and Females with Gastrointestinal Cancers
Source: Int J Mol Sci. 2023 Jun 29;24(13):10847. doi: 10.3390/ijms241310847 (PMC10342094; doi:10.3390/ijms241310847)
Supplement: Supplementary file 1 [file ijms-24-10847-s001.zip › ijms-2430576-supplementary.pdf]

Supplementary Figure S1. Gene set enrichment analysis reveals differential pathway enrichment in obese CD8+ and CD14+ cells. GSEA enriched for several immune (interferon, INFA, IFNB, etc.), metabolic pathways (oxidative phosphorylation, oxidized phospholipids, etc.) in both the TCGA's A) COAD and B) ESCA.

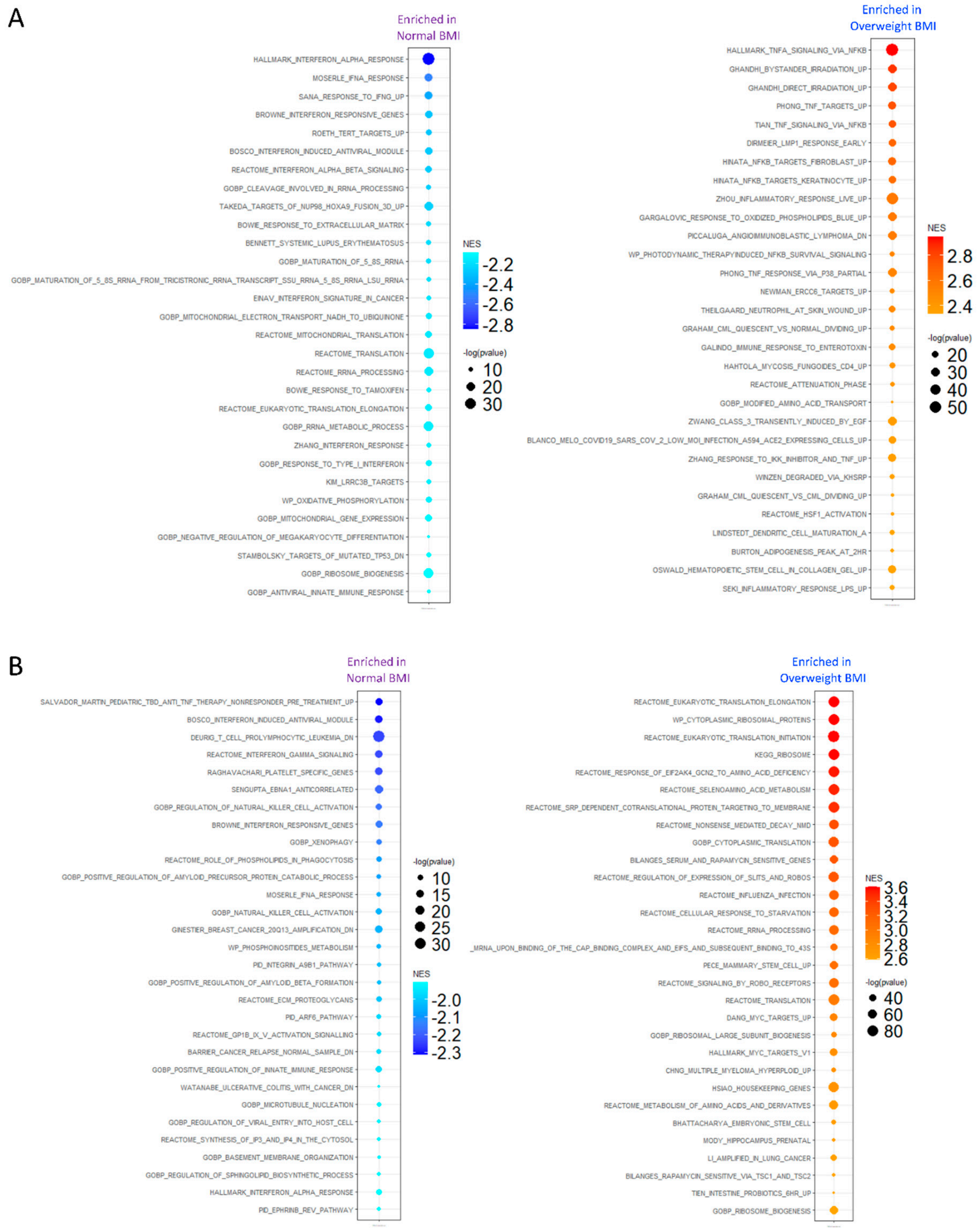

Supplementary Figure S2. Differential predicted response to immunotherapy in CD8+ and CD14+ cells. Modeling of CD8+(left) and CD14+ (right) pathways associated with response to immunotherapy revealed differential response in obese vs. non-obese patients. In the context of obesity, both CD8+ and CD14+ cells decreased expression (blue triangle) of CTLA4 and PIK family genes. Further, both seemed to upregulate (red triangle) CD28, albeit to different extents. However, only CD8+ cells upregulated CD80 in obese patients, as compared to non-obese patients.

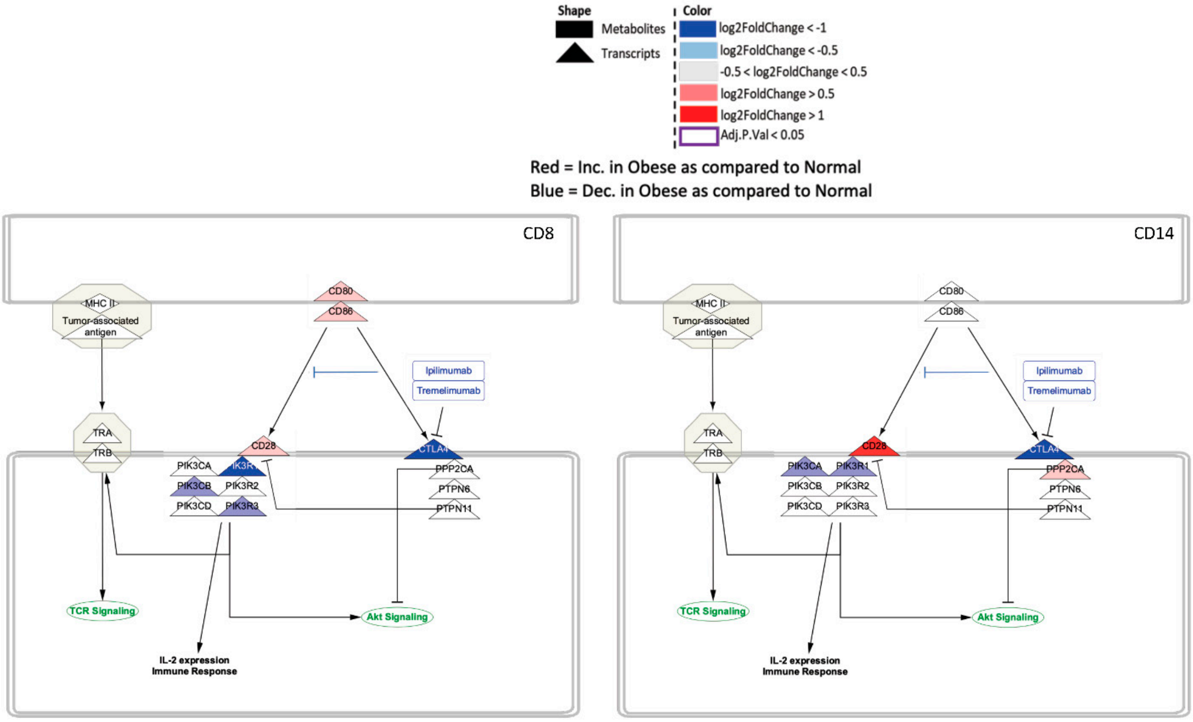

Supplementary Figure S3. Differential gene expression analysis of COAD male and female obese vs. non-obese patients highlights varying transcriptional difference. A) Male COAD assessment resulted in many differentially expressed genes DEGs; adj. p < 0.05, |logFC|>1.5, red) when comparing patients with BMI considered normal (left, light blue) to those considered overweight (right, dark blue). B) Female COAD assessment resulted in a large number of differentially expressed genes (adj. p < 0.05, |logFC|>1.5, red) when comparing patients with BMI considered normal (left, light pink) to those considered overweight (right, dark pink).

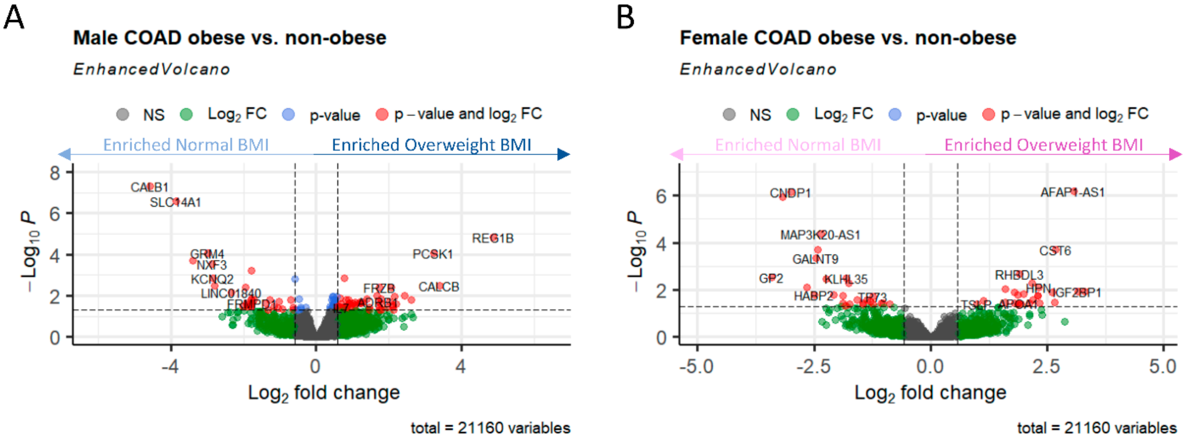

Supplementary Figure S4: Study Workflow. In this study, we first A) assessed consortium GI adenocarcinoma transcriptomic sequencing datasets from ORIEN and TCGA. Comparing obese and non-obese individuals, these datasets were then scrutinized via DEG, GSEA and metabolic pipeline analysis to understand and predict metabolic dysregulation associated with obesity. B) To confirm findings from these large consortia data, sorted CD8+ and CD14+ PBMCs and serum of patients treated at our institute were utilized for paired metabolomics (serum) and transcriptomics (PBMCs), to reveal convergence of metabolic dysregulation both in the context of obesity and sex, which was useful for metabolic and immune pathway development.

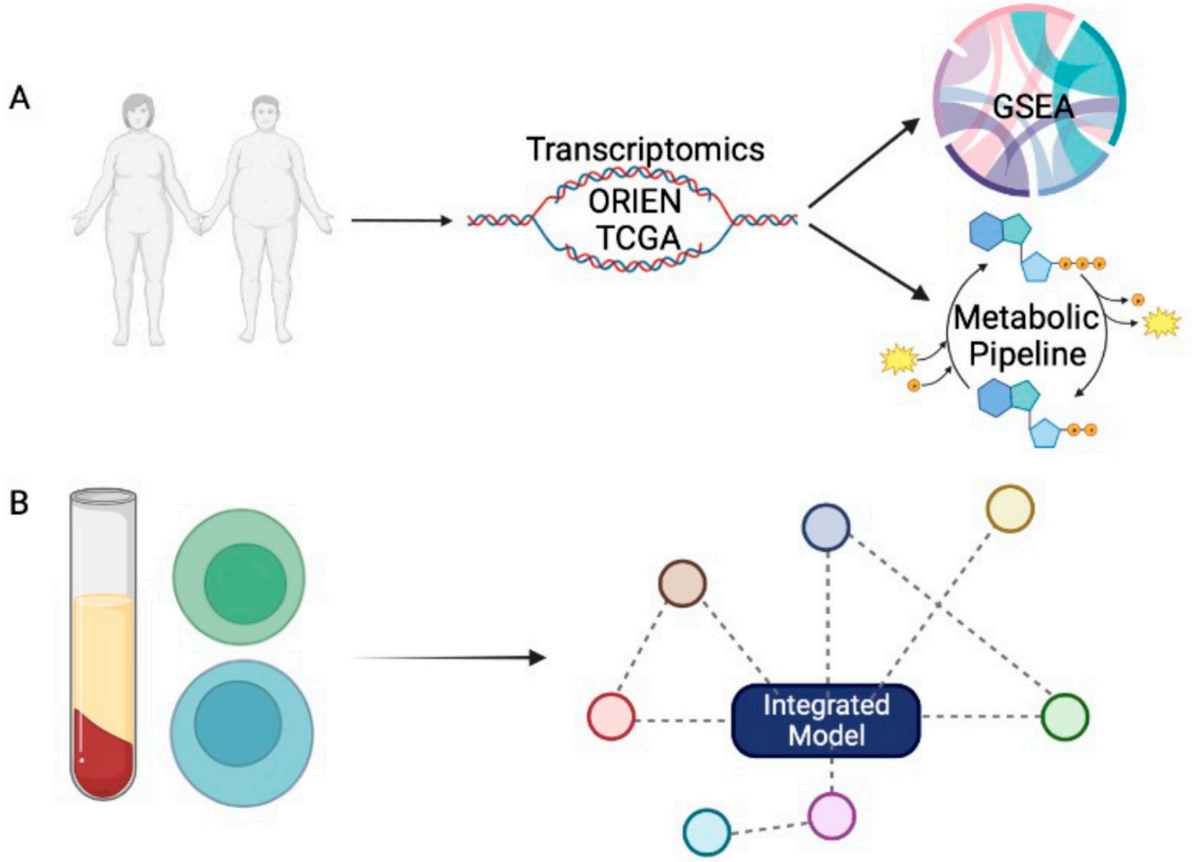

Supplementary Table S1: The demographic and clinical characteristics of the internal cohort

Age	Median/Min/Max	67/46/79
Body Mass Index (BMI)	Median/Min/Max	24.3/21/3/30.7
Gender	Male	5 (83.0%)
	Female	1 (17.0%)
Immune Checkpoint Inhibitor	Pembrolizumab	4 (67.0%)
	Nivolumab	2 (33.0%)
Race	Black	1 (17%)
	White	5 (83%)
Tumor location	Gastroesophageal junction	3 (50%)

	Esophageal	2 (33.3%)
	Gastric	1 17.0%)
	Sigmoid colon	1 (8.3%)
Histology	Adenocarcinoma	5 (83%)
	Squamous cell Cancer	1 (8.3%)
